# Infancy and childhood growth and physical activity in adolescence: prospective birth cohort study from Brazil

**DOI:** 10.1186/1479-5868-9-82

**Published:** 2012-07-02

**Authors:** Pedro C Hallal, Samuel C Dumith, Ulf Ekelund, Felipe F Reichert, Ana M B Menezes, Cesar G Victora, Jonathan C K Wells

**Affiliations:** 1 Federal University of Pelotas, Rua Marechal Deodoro 1160, 96020-220, Pelotas, Brazil; 2 MRC Epidemiology Unit, Institute of Metabolic Science, Addenbrookes Hospital, Box 285, Cambridge, CB2 0QQ, UK; 3 Childhood Nutrition Centre, Institute of Child Health, 30 Guilford Street, WC1N 1EH, London, UK

**Keywords:** Motor activity, Exercise, Epidemiology, Prospective studies, DOHaD

## Abstract

**Background:**

The Developmental Origins of Health and Disease hypothesis suggests that intrauterine, infancy and early childhood variables play a key role at programming later health. However, little is known on the programming of behavioral variables, because most studies so far focused on chronic disease-related and human capital outcomes. The aim of the present study was to evaluate the effects of prenatal, infancy and childhood weight and length/height gains on objectively-measured physical activity (PA) in adolescence.

**Methods:**

This is a prospective birth cohort study in Pelotas, Brazil, including 457 adolescents (mean age: 13.3 years) with weight and length/height data at birth, one, three and six months, one and four years of age. PA was measured using a GT1M Actigraph accelerometer, and expressed as (a) minutes per day spent on sedentary, light, moderate, vigorous and very-vigorous activities; (b) total counts per day.

**Results:**

61.3% of the adolescents accumulated 60+ minutes of moderate-to-vigorous PA per day. Weight and length/height trajectories in infancy and childhood were similar between those classified as active or inactive at 13.3 years. However, those classified as inactive were heavier and taller at all ages; differences were statistically significant only in terms of length at three, six and 12 months.

**Conclusions:**

Weight gain in infancy and childhood did not predict variability in adolescent PA, but those active in adolescence showed somewhat smaller average gains in length in infancy. These findings suggest that PA may partially be sensitive to early hormonal programming, or that genetic factors may affect both early growth and later metabolism or predisposition for PA.

## Introduction

The Developmental Origins of Health and Disease [1] (DOHaD) hypothesis suggests that intrauterine, infancy and early childhood variables play a key role at programming later health. However, little is known on the programming of behavioral variables, because most studies so far focused on chronic disease-related (blood pressure, glucose, coronary heart disease) and human capital (schooling, height, income) outcomes [2-9]. Previous birth cohort studies from Brazil and UK examined early life determinants of physical activity [10,11], and found that social variables were more strongly related to later physical activity than biological determinants. A recent meta-analysis from four studies in which objectively-measured data on physical activity were available observed no consistent effect of birthweight on later physical activity [12].

Little is known on the effects of prenatal, infancy and early childhood growth on later physical activity. Of particular interest is the possibility that trajectories of weight and length/height might have different influences on later physical activity. Due to the compelling evidence linking rapid weight gain, particularly after the age of two years [9], with later non-communicable disease risk, we aimed to test whether or not reduced physical activity practice among those growing fast would be part of this pathway. The aim of the present study was to evaluate the effect of prenatal, infancy and childhood weight and length/height gains on objectively-measured physical activity in adolescence.

## Methods

### Participants

Mothers of all children (N = 5,265) born in 1993 in the city of Pelotas, Southern Brazil, were invited to take part in a birth cohort study [13,14]. All but 16 agreed to participate. A group of 511 cohort participants was seen at the mean ages of one, three and six months, one, four, and 13.3 years of age. The Federal University of Pelotas Medical School Ethics Committee approved all phases of the 1993 Pelotas (Brazil) Birth Cohort Study. Parents or guardians signed informed consents forms prior to each follow up wave.

### Measurements

Physical activity at 13.3 years was measured using a GT1M Actigraph accelerometer. Monitors were attached to the hip for three to five days. A minimum of 10 hours per day of activity recording was required for being included in this analysis. An epoch of 1 second was used and subjects were included in the analyses if providing at least two full days of accelerometer data (>95% of the participants provided data on three or more days). Periods of 60 or more minutes of consecutive zeros were treated as non-use. We analyzed two physical activity variables in this study: total counts per day as an indicator of overall physical activity and minutes per day spent in sedentary (0–100 counts), light (101–2000 counts), moderate (2001–5000 counts), vigorous (5001–8000 counts) and very vigorous activities (>8000 counts). We additionally estimated the proportion of adolescents practicing 60+ minutes per days in moderate-to-vigorous physical activity (MVPA). Details on the accelerometer data collection and handling are available elsewhere [15].

Birth weight and length were measured at the hospital using standardized procedures by the research team [16]. In all subsequent follow up visits, anthropometric measurements were taken at the cohort member’s household. Standardized equipment was used by trained personnel. Up to the age of two years, length was measured. From this point onwards, standing height was measured. For this reason, we use “length/height” throughout the article.

### Statistics

Weight and length/height measured at different ages are strongly correlated, leading to problems with collinearity [3]. We therefore modeled the relationship between early weight and length/height and adolescent physical activity using conditional variables. For each time point in infancy and childhood, conditional weight and length/height were calculated as the residual from linear regression of weight in kg (or length/height in cm) at that age on any prior weights (or lengths/heights). The residuals are therefore uncorrelated with any prior weight (or length/height) measures. These conditional variables may be interpreted as the deviation from the preceding growth interval in weight (or length/height) predicted by birthweight (or length) and any prior weights (or lengths/heights) when analyzed in linear regression models.

Our analyses initially compared individuals from the subsample included in this study with the full cohort. We then analyzed Z-score weight and length/height trajectories of adolescents classified as active at age 13 years, based on the 60 minutes per day of MVPA threshold, as compared to those inactive. Finally, we use linear regression models in which accelerometer counts is the outcome variable and conditional weight and length/height are the main exposures. The outcome variable was normally distributed, with a slight asymmetry to the right. We run unadjusted and adjusted analyses, incorporating adjustment for sex, gestational age, family income, maternal schooling, maternal body mass index, maternal smoking during pregnancy, and all other conditional weight and length/height variables. We repeated all models after log-transforming the outcome variable, but because results were consistent with those obtained using the non-transformed variable, we opted to keep only the more simple approach in the article. Final models were tested for collinearity (using the command “*vif*” in Stata 11), and no evidence of such a problem was detected.

## Results

The subsample included in this analysis is similar to the full cohort in terms of most variables of interest (Table 1). The only statistically significant difference is the proportion of males in the two samples, explained by a higher likelihood of losses to follow up among boys than girls. Physical activity levels estimated from self-report at 11 years are virtually identical between the subsample and the full cohort. The mean MVPA was 72.6 minutes per day (median 67.4; SD 31.5). The proportion of adolescents accumulating 60 minutes or more per day of MVPA was 61.3% (69.8% among boys and 51.2% among girls; p < 0.001). Boys participated, on average, in 12 minutes per day of vigorous or very vigorous physical activity, whereas girls accumulated seven minutes per day in this intensity (Table 2). The amount of time (min/day) spent in sedentary activities was identical between boys and girls (P = 0.97). Because results of sex-stratified analyses were similar, all results are presented for both sexes combined.

**Table 1:** Comparison between the subsample included in the present analyses (N=457) and the remaining cohort members (N=4,792) in terms of sociodemographic and anthropometric characteristics and self-reported physical activity at 11 years of age

**Variable**	**Subsample**	**Full cohort**
% males	52.1%	48.9%
% mothers with no schooling	2.8%	2.5%
% obese mothers (≥ 30 kg/m^2^)	2.8%	2.5%
% low birthweight (<2500 g)	8.3%	9.9%
% preterm (<37 weeks)	9.3%	11.6%
% firstborns	30.9%	35.5%
% active at 11 years by self-report (≥ 300 min/wk)	62.5%	62.7%

**Table 2:** Objectively-measured physical activity patterns at 13.3 years among boys and girls (N=457)

**Variable**	**All Mean (SD)**	**Boys Mean (SD)**	**Girls Mean (SD)**	**P value**
Time spent in different intensities of physical activity (min/day)				
Sedentary (0-100 counts)	660 (80)	660 (82)	661 (78)	0.97
Light (101-2000 counts)	189 (45)	200 (48)	177 (37)	<0.001
Moderate (2001-5000 counts)	63 (27)	69 (28)	58 (25)	<0.001
Vigorous (5001-8000 counts)	8 (6)	10 (7)	6 (5)	<0.001
Very vigorous (> 8000 counts)	1 (2)	2 (2)	1 (2)	<0.001
% active (≥ 60 min/day of moderate-to-vigorous physical activity)	61.3%	69.8%	52.1%	<0.001

**Figure 1:**
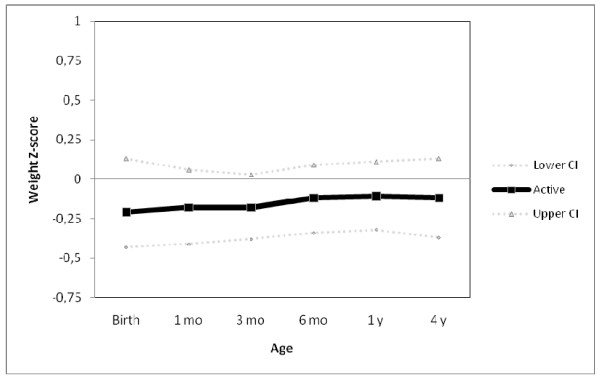
**Weight trajectories of individuals classified as active (≥ 60 min/day of moderate-to-vigorous physical activity) at 13.3 years as compared to those inactive (zero line).** N=457.

**Figure 2:**
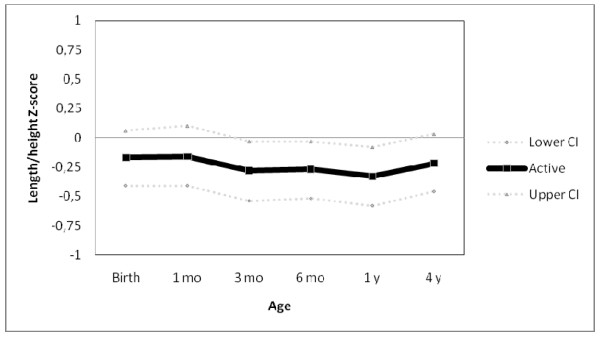
**Length/height trajectories of individuals classified as active (≥ 60 min/day of moderate-to-vigorous physical activity) at 13.3 years as compared to those inactive (zero line).** N=457.

Figure 1 shows weight trajectories of adolescents classified as active or inactive at 13.3 years according to the 60 minutes per day of MVPA threshold. The horizontal line at the zero mark in the y axis represents non-active subjects, who comprise 38.7% of the cohort, against which the mean and 95% confidence interval of active subjects are plotted. At all ages, those who were classified as active at 13.3 years were slightly lighter than those categorized as inactive, although all confidence intervals include the unity. In terms of length/height trajectories (Figure 2) active subjects tended to be shorter at all ages, with confidence intervals excluding the unity at 3, 6 and 12 months.

Table 3 shows unadjusted and multivariable models using counts per day as the outcome variable, with weight and length expressed as conditional z-scores. In the adjusted models, conditional weights at different ages was largely unrelated to later physical activity, whereas conditional lengths at 3 and 12 months were inversely related to adolescent physical activity levels. Some important differences between unadjusted and adjusted results were found, most of which due to the inclusion of sex and socioeconomic indicators (income and education) in the models. This is explained by the fact that sex and socioeconomic position are associated with both growth patterns and adolescent physical activity.

**Table 3:** Objectively-measured physical activity levels at 13.3 years (counts) according to weight and length/height trajectories: linear regressions analysis (N=457)

**Variable**	**Unadjusted analysis**		Adjusted analysis*	
**Weight**	**Coefficient (95%CI)**	**P**	**Coefficient (95%CI)**	**P**
Birth	-8.6 (-20.5; 3.2)	0.15	-13.1 (-32.9; 6.6)	0.19
1 month	-6.0 (-19.4; 7.5)	0.38	-2.8 (-19.7; 14.1)	0.89
3 months	-11.9 (-25.8; 2.2)	0.10	2.9 (-12.7; 18.4)	0.72
6 months	-2.0 (-16.4; 12.4)	0.78	8.1 (-6.8; 23.1)	0.29
1 year	-6.4 (-21.5; 8.7)	0.38	4.5 (-10.7; 19.6)	0.56
4 years	-15.5 (-30.7; -0.2)	0.05	-8.6 (-27.1; 10.0)	0.37
Length/height				
Birth	-9.1 (-20.3; 2.1)	0.11	5.9 (-11.7; 23.5)	0.51
1 month	-5.7 (-19.0; 7.6)	0.40	8.2 (-10.6; 27.0)	0.39
3 months	-20.6 (-34.5; -6.7)	0.01	-18.0 (-33.0; -2.9)	0.02
6 months	-11.7 (-25.4; 2.0)	0.09	-11.3 (-26.0; 6.4)	0.13
1 year	-16.3 (-29.9; -2.8)	0.02	-23.4 (-39.7; -7.4)	0.01
4 years	-3.0 (-19.8; 13.9)	0.73	12.5 (-5.0; 30.1)	0.16

* Adjusted for sex, gestational age, family income, maternal schooling, maternal body mass index, maternal smoking during pregnancy, and all other weight and length/height variables.

## Discussion

Physical activity is likely determined by a complex mixture of biological, social, cultural and environmental factors. Most studies so far have focused on sociodemographic factors, features of the built environment and social support [17]. Studies of physical activity in the DOHaD context are still rare [10-12,18,19]. In a prospective birth cohort study in Brazil, we evaluated the association between early growth, both in terms of weight and length/height, and objectively-measured physical activity. In summary, we found similar patterns of weight gain between those classified as active or inactive at 13.3 years of age, thus suggesting that prenatal, infancy and childhood weight gains are not major determinants of physical activity levels in adolescence in our cohort; however we also observed subtle differences in early length gain patterns between active and sedentary adolescents, and this observation offer a preliminary insight into a topic meriting further research.

Results on the association between birth-weight and physical activity are still inconclusive. Some studies reported lower motor skills and reduced aerobic fitness among those born with very low birthweight [20], but studies in population-based samples have, in general, failed to detect associations between birthweight and physical activity [12]. Studies from low and middle-income countries have consistently reported that adequate birthweight and rapid weight and length gains in the first two years of life are associated with increased human capital, and does not increase – or even reduces - the risk of most precursors for chronic diseases [9]. Rapid weight gain after the age of two years, however, is consistently associated with the later appearance of risk factors for chronic diseases [21-23]. The null findings we report here suggest that reduced physical activity levels in adolescence are not part of the pathway leading from early growth patterns to later health.

Analyses comparing the early growth trajectories of subjects experiencing chronic diseases as adults [24] are useful but differences between groups may appear small in part because other subsequent exposures have diluted the magnitude of the effect. Nevertheless, our results suggest that there is a likely link between length gain in infancy and subsequent physical activity practice, and these findings were confirmed by the conditional growth analyses.

Some limitation of the present study should be mentioned. We do not have data at the age of two years, a well-known threshold in terms of length gain [25]. Puberty status was not taken into account, but it is known that it can influence adolescent health and development. Although our subsample is comparable to the full cohort in terms of some key variable, the possibility of some degree of selection bias cannot be ruled out.

If our results on the association between length gain in infancy and later physical activity are confirmed by others, one possible explanation is that adolescent physical activity is affected by early hormonal programming. Early growth in height is associated with IGF-1 levels. Faster growth in infancy implies higher IGF-1, which might have effects on later metabolism. An alternative explanation is that there may be subtle genetic differences, in terms of genes which affect both early growth and later physical activity. For example, Elks and colleagues found that a proportion of the genes associated with adult obesity were also associated with early growth patterns [26].

## Conclusions

Early weight gain does not seem to be a strong predictor of adolescent physical activity in this population. However, length gain in infancy seems to play a role at determining adolescent physical activity. Further studies are required in different populations due to the paucity of data on this association.

## Competing interests

The author(s) declare that they have no competing interests.

## Authors’ contributions

PC Hallal had the original idea and led writing of the manuscript. SC Dumith and FF Reichert led the data analyses. U Ekelund was responsible for all accelerometry data, and JC Wells was responsible for all body composition data. AM Menezes and CG Victora coordinate the 1993 Pelotas cohort. All authors commented on drafts of the manuscript, suggested changes and approved the final version.
